# Evaluating a questionnaire to measure improvement initiatives in Swedish healthcare

**DOI:** 10.1186/1472-6963-13-48

**Published:** 2013-02-07

**Authors:** Ann-Christine Andersson, Mattias Elg, Kent-Inge Perseius, Ewa Idvall

**Affiliations:** 1 Division of Quality Technology and Management, Linköping University, Linköping, Sweden; 2 Development Department, Kalmar County Council, Kalmar, Sweden; 3 Faculty of Health and Society, Malmö University, Malmö, Sweden; 4 Division of Quality Technology and Management and HELIX Vinn Excellence Centre, Linköping University, Linköping, Sweden; 5 Nyckeln competence centre for pedagogy in health care, Kalmar County Council, Kalmar, Sweden; 6 the Department of Neurobiology, Caring Sciences and Society, Karolinska Institutet, Stockholm, Sweden; 7 Faculty of Health and Society, Malmö University, Malmö, Sweden; 8 Department of Intensive Care and Perioperative Medicine, Skåne University Hospital, Malmö, Sweden; 9 Box 601, SE-391 26, Kalmar, Sweden

**Keywords:** Questionnaire development, Psychometric test, Improvement measurements, Improvement program, Questionnaire assessment, Healthcare setting

## Abstract

**Background:**

Quality improvement initiatives have expanded recently within the healthcare sector. Studies have shown that less than 40% of these initiatives are successful, indicating the need for an instrument that can measure the progress and results of quality improvement initiatives and answer questions about how quality initiatives are conducted. The aim of the present study was to develop and test an instrument to measure improvement process and outcome in Swedish healthcare.

**Methods:**

A questionnaire, founded on the Minnesota Innovation Survey (MIS), was developed in several steps. Items were merged and answer alternatives were revised. Employees participating in a county council improvement program received the web-based questionnaire. Data was analysed by descriptive statistics and correlation analysis. The questionnaire psychometric properties were investigated and an exploratory factor analysis was conducted.

**Results:**

The Swedish Improvement Measurement Questionnaire consists of 27 items. The Improvement Effectiveness Outcome dimension consists of three items and has a Cronbach’s alpha coefficient of 0.67. The Internal Improvement Processes dimension consists of eight sub-dimensions with a total of 24 items. Cronbach’s alpha coefficient for the complete dimension was 0.72. Three significant item correlations were found. A large involvement in the improvement initiative was shown and the majority of the respondents were satisfied with their work.

**Conclusions:**

The psychometric property tests suggest initial support for the questionnaire to study and evaluate quality improvement initiatives in Swedish healthcare settings. The overall satisfaction with the quality improvement initiative correlates positively to the awareness of individual responsibilities.

## Background

The number of quality improvement initiatives has increased within the healthcare sector in recent decades. These efforts to improve quality can be seen as a response to demands for more cost-effectiveness and better medical results. However, studies have shown that less than 40% of these initiatives are successful [1]. The reason why specific improvement initiatives in healthcare fail or succeed is, therefore, a central question in studies of planned change. The need for more evidence about how to organize and manage new quality initiatives is identified as an important task within studies of healthcare improvement [1,2].

Surveys are a frequently used measurement tool in healthcare settings [3]. Surveys for assessment of innovation climate and innovation cultures have been developed. One of those is the Creative Climate Questionnaire (CCQ) aiming to measure the climate in an organization regarding creativity and innovations [4]. This survey was recently used in a study of implementation of new tools in primary healthcare centres [5]. Staff and management were shown to differ in their assessment of the organizational climate at the unit, with managers scoring higher. Olsson et al. [6] developed a model (the Swedish OCM) connected with a survey to predict outcomes of organizational change. Their model is directed towards predicting success or failure within a change project. A European project (MARQuIS) developed a web-based survey to measure quality improvement strategies in acute care hospitals in the European Union [7]. The survey consisted of four sections, one at an overall hospital quality improvement level, the other three on quality management for medical conditions (acute myocardial infarctions, acute appendicitis and deliveries). The most broadly used strategies were related to external assessments, such as ISO, while activities related to patient involvement were implemented less often.

To the best of our knowledge, there are no surveys today in a Swedish context that can answer questions about how quality improvement initiatives are conducted and develop within Swedish healthcare. Wanting to contribute to the understanding of the development of quality improvement initiatives in Swedish healthcare by conducting longitudinal studies on improvement initiatives, we needed a measurement mechanism. The Minnesota Innovation Survey (MIS) was found to be a comprehensive survey including different dimensions of innovations and at the same time was developed to measure over time [8]. It has a broad focus on change processes as well as antecedents and motivations for/motors of change. Therefore the MIS survey, with its focus on investigating aspects of change, could serve as a foundation to develop an instrument to measure improvements. The aim of the present study was to develop and test an instrument, based on MIS, to measure how improvement processes develop in a Swedish healthcare context.

## Methods

### The county council improvement program – the empirical context

The quality improvement program started in 2007 and was a political investment with the vision and aim to make the county council a learning organization with welfare of patients in focus. The program is implemented both from the top management level and at individual departments/clinics/primary healthcare centres. The program is described on the county council website [9]. One of the activities is the methodological guided improvement program inviting staff teams to work with an innovation idea in a program using the Breakthrough Series Collaborative methodology and supervisors/facilitators [10]. As of autumn 2010 six of those programs had been started, involving about 130 teams and 610 staff members, the latest including staff and teams from some of the municipalities within the county council.

### Minnesota innovation survey

**Figure 1:**
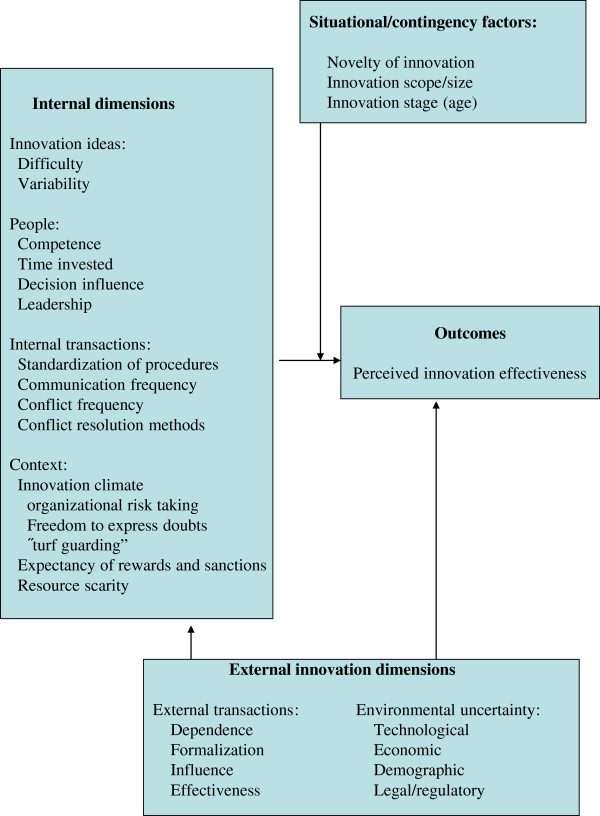
**Dimensions in measurement model of Minnesota Innovation Survey.** Source: Van de Ven *et al*. (2000), p. 56.

The Minnesota Innovation Survey (MIS) was developed to measure how innovations emerge, develop, grow or terminate over time [8]. MIS is a comprehensive instrument consisting of 102 items including partial and open-ended questions. In addition there are ten categories of demographic data. It has been used in different contexts, both in manufacturing and in service operations, such as medical practices, public schools, data-processing technology and hospital organizations. The survey is built on a theory of innovation management that encompasses five basic concepts: ideas, people, transactions, context, and outcomes. These concepts are seen as central factors concerning management of innovation processes. The conceptual base framework consists of dimensions grouped into four clusters: Internal dimensions, Situational/contingency factors, External innovation dimensions and Outcomes (Figure 1). Different psychometric tests showed evidence for convergent validity as well as discriminant validity [8]. The MIS survey was found to be too extensive for our purposes, but two of the dimensions were suitable, Perceived Innovation Effectiveness (n=5) measuring the outcome, and Internal Dimensions (n=32) measuring the development processes. Therefore those two dimensions were used as a foundation in developing our questionnaire.

### The Swedish Improvement Measurement Questionnaire (SIMQ)

Permission was granted from the creators of the original MIS to use it as a foundation in developing a Swedish instrument to measure improvements. Extensive modification and development of the two suitable dimensions of the MIS was done in several steps. Research team meetings were held between the different steps and during this process different ways to validate the questionnaire were applied. The original survey in English was translated into Swedish by a professional translator. Literally translated words and/or sentences were edited into more idiomatic Swedish and words were adapted to fit the Swedish healthcare and county council organization.

A focus group discussion was conducted with five former participants from earlier improvement programs. The focus group interview was tape recorded and notes were taken. Following the focus group’s suggestions, some questions were paraphrased and others reworded. The answers and answer scales were revised and sometimes changed. Individual interviews were held with three members of improvement program support staff. Their views and ideas were considered in a new revision, some questions were merged and others were moved. An alternative (“Do not know”) was added to some questions.

An expert in quality management was then asked to review the revised questionnaire. Based on his suggestions a revision was made, items were removed and merged, and the questionnaire was shortened. Even if the changes made were too extensive to be able to use the new questionnaire as comparison, a back translation was conducted, by another translator, to compare that the two dimensions were still present. An introductory text was written to suit the study and Swedish context, and to fit the web layout. The questionnaire was entered into the web-based survey program esMaker NX2. An answer was required to all items with fixed scales; otherwise it was not possible to proceed with the questionnaire.

### Background data

Background data consisted of demographic data about the participants, such as profession, gender and years of work experience. Other background data covered respondent’s previous experiences in improvement work and the time spent on different activities within the improvement work.

### Participants and data collection

The present study contains data from two improvement programs, ongoing from September 2009 until November 2010. Employees within the county council (n=171), the municipalities (n=39), in all 210 participants in the two improvement programs received the survey. The participants received written and verbal information from the first author at the regular improvement program meetings. The survey was sent by e-mail and the informants consented by answering the web-based questionnaire. After one week and two weeks reminders were sent automatically to those who had not answered. The survey study was conducted according to general ethical standards and approved by the Regional Ethics Review board in Linköping, Sweden (Dnr 179–09).

### Data analysis

Data was analyzed using Statistica version 8.0 (StatSoft, Tulsa, OK, USA). Descriptive statistics are presented in both actual frequencies and as percentages, range, mean, standard deviation (SD) and median. The questionnaire’s psychometric properties were investigated through internal consistency scale analysis and an exploratory factor analysis was conducted for the total dimensions as well as each sub-dimension containing more than one item. Cronbach’s alpha coefficients are presented at both dimension and sub-dimension levels and the floor/ceiling effect in percentages are presented for each item. At both dimension and sub-dimension level the number of items needed to reach a Cronbach' s alpha 0.7 was calculated. Correlations were calculated between the two dimensions. In addition participants’ written free comments are presented.

## Results

### The Swedish Improvement Measurement Questionnaire (SIMQ)

The developed questionnaire consisted of two dimensions, “Improvement Effectiveness Outcome” (n=3) and “Internal Improvement Processes” (n=24), the latter divided into eight sub-dimensions (items are presented fully written out in Table 1). The items consisted of both questions and statements, and were answered in a verbal five-point scale, from “Very little” to “Very much” or “Not at all” to “A lot”. There were items with different scales, such as “Absolutely do not agree” to “Absolutely agree”. In some items the answer alternative “Do not know” was added. Likewise, some items had the possibility to write comments.

**Table 1:** Results from the dimensions “Improvement effectiveness outcome” and “internal improvement processes” 27 items, n (%)

**Improvement effectiveness outcome (n=92)**								
	**Not at all (0)**	**A little (1)**	**Some (2)**	**Quite a bit (3)**	**A lot (4)**	**Mean (SD)**		**Median**
22. Overall, how satisfied are you with the progress that has been made in the work to develop the improvement idea during the past month	1 (1)	5 (6)	16 (17)	51 (55)	19 (21)	2.9 (0.8)		Quite a bit
24. How much does the improvement idea contribute to improving the work at your unit?	2 (2)	6 (7)	32 (35)	27 (29)	25 (27)	2.7 (1.0) / 3		Quite a bit
	**Far below (0)**	**Somewhat below (1)**	**As expected (2)**	**Somewhat above (3)**	**Far above (4)**	**Mean (SD)**		**Median**
23. To what extent is your progress with the improvement idea below or above your original expectations?	2 (2)	6 (7)	45 (49)	35 (38)	4 (4)	2.4 (0.8)		As expected
**Internal Improvement Processes (8 sub dimensions)**								
***Improvement Uncertainty*** (n=92)	**Very easy (0)**	**Quite easy (1)**	**Moderate (2)**	**Quite difficult (3)**	**Very difficult (4)**	**Mean (SD)**		**Median**
2. How easy is it for you to know ahead of time what steps are necessary to develop the improvement idea?	1 (1)	35 (38)	45 (49)	11 (12)	0	1.7 (0.7)		Moderate
	**Not at all (0)**	**Once (1)**	**Every other week (2)**	**Every week (3)**	**Every day (4)**	**Mean (SD)**		**Median**
7. How often in the past month did problems arise during development of the improvement idea?	42 (46)	27 (29)	13 (14)	9 (10)	1 (1)	0.917 (1.0) /1		Once
***Resource scarcity*** (n=92)								
How much must your improvement idea compete with other activities within your unit, when it comes to	**Not at all (0)**	**Little (1)**	**Some (2)**	**Quite a bit (3)**	**A lot (4)**	**Mean (SD)**		**Median**
35a. Economic resources?	42 (46)	23 (25)	16 (17)	7 (8)	4 (4)	1.0 (1.2)		Little
35b. Material, space, and equipment?	48 (52)	21 (23)	16 (17)	6 (7)	1 (1)	0.8 (1.0)		Not at all
35c. Attention from the executive level?	31 (34)	26 (28)	16 (17)	14 (15)	5 (5)	1.3 (1.2)		Little
35d. Personnel?	22 (24)	21 (23)	23 (25)	17 (18)	9 (10)	1.7 (1.3)		Some
35e. Time to work with the improvement idea?	4 (4)	16 (17)	24 (26)	28 (30)	20 (22)	2.5 (1.1)		Quite a bit
***Standardization of procedures*** (n=92)	**Very little (0)**	**Little (1)**	**Moderate (2)**	**Much (3)**	**Very much (4)**	**Mean (SD)**		**Median**
3. To what extent is your work on the improvement idea supported by the methods used in the improvement program?	1 (1)	8 (9)	42 (46)	38 (41)	3 (3)	2.4 (0.7)		Moderate
***Expectations of Rewards and Sanctions*** (n=92)								
How likely is it that the following will occur if the goals of the improvement idea have been achieved:	**Not likely (0)**	**Hardly likely (1)**	**Likely (2)**	**Very likely (3)**	**Totally likely (4)**	**Mean (SD)**		**Median**
15a. Everyone involved, as a group, will be rewarded or recognized for their collective efforts	9 (10)	16 (17)	29 (32)	27 (29)	11 (12)	2.2 (1.2)		Likely
15b. Only some participants will be rewarded or recognized for their individual efforts	42 (46)	37 (40)	8 (9)	5 (5)	0	0.7 (0.8)		Hardly likely
How likely is it that the following will occur if the goals of the improvement idea have not been achieved:								
16a. Everyone involved, as a group, will be reprimanded or told to “shape up” to improve their efforts.	27 (29)	34 (37)	20 (22)	9 (10)	2 (2)	1.2 (1.0)		Hardly likely
16b. Only some participants will be reprimanded or told to “shape up” to improve their efforts	47 (51)	37 (40)	8 (9)	0	0	0.6 (0.6)		Not likely
***Improvement Group Leadership*** (n=92)	**Absolutely do not agree (0)**	**Mostly do not agree (1)**	**Neutral (2)**	**Mostly agree (3)**	**Absolutely agree (4)**	**Mean (SD)**		**Median**
10. The project leader of the improvement idea encourages the participants to take initiative	1 (1)	2 (2)	18 (20)	34 (37)	37 (40)	3.1 (0.9)		Mostly agree
11. The participants involved in the improvement idea are aware of their individual responsibilities	0	1 (1)	7 (8)	46 (50)	38 (41)	3.3 (0.7)		Mostly agree
12. The project leader for the improvement idea places great emphasis on getting the work done.	1 (1)	1 (1)	15 (16)	36 (39)	39 (42)	3.2 (0.8)		Mostly agree
13. The project leader has great confidence in the participants involved in the improvement idea	0	1 (1)	14 (15)	26 (28)	51 (55)	3.4 (0.8)		Absolutely agree
	**Not at all (0)**	**Little (1)**	**Some (2)**	**Quite a bit (3)**	**A lot (4)**	**Mean (SD)**		**Median**
21. Do those involved in working with the improvement idea receive feedback from “improvement support”/their supervisor on how they can improve their work?	2 (2)	12 (13)	31 (34)	29 (32)	18 (19)	2.5 (1.0)		Quite a bit
***Freedom to Express Doubts*** (n=92)	**Absolutely do not agree (0)**	**Mostly do not agree (1)**	**Neutral (2)**	**Mostly agree (3)**	**Absolutely agree (4)**	**Mean (SD)**		**Median**
14. To avoid causing disharmony I often feel I cannot say what I think about the work on the improvement idea.	60 (65)	17 (18)	7 (8)	6 (7)	2 (2)	0.6 (1.0)		Absolutely do not agree
***Learning Encouragement*** (n=92)	**Absolutely does not apply (0)**	**Mostly does not apply (1)**	**Neutral (2)**	**Mostly apply (3)**	**Absolutely applies(4)**	**Mean (SD)**		**Median**
33. If a colleague tries something new and fails, this is viewed as something that could harm her/his future career in the county council.	31 (34)	24 (26)	36 (39)	1 (1)	0	1.1 (0.1)		Mostly does not apply
34. The county council prioritizes experimenting with new ideas.	5 (5)	11 (12)	54 (59)	20 (22)	2 (2)	2.0 (0.8)		Neutral
***Decision Influence***								
How much influence have you had on each of the following decisions that might have been made during the past month?	No decision made * (0)	**None (1)**	**Little (2)**	**Some (3)**	**Quite a bit (4)**	**A lot (5)**	**Mean (SD)**	**Median**
6a. Preparing goals and measures for the improvement idea? (n=91)	1 (1)	0	6 (6)	7 (8)	43 (47)	35 (38)	4.2 (0.8)	Quite a bit
6b. Deciding which activities should be carried out within the improvement idea? (n=90)	2 (2)	0	4 (4)	8 (9)	42 (46)	36 (39)	4.2 (0.8)	Quite a bit
6c. Deciding on economic funds and resources for the improvement idea? (n=64)	28 (30)	39 (42)	13 (14)	6 (7)	2 (2)	4 (4)	1.7 (1.2)	None
6d. Recruiting colleagues to work with the improvement idea? (n=71)	21 (23)	21 (23)	9 (10)	10 (11)	18 (20)	13 (14)	2.9 (1.5)	Some

* Answer alternative “No decision made” are excluded from mean (SD) and Median calculations.

### Participants

Response rates divided into professions are presented in Table 2. The response rate from county council employees was 45% (n=77) and from municipal employees 38% (n=15). Seven of the twelve municipalities within the county council were represented. A total of 44% (n=92) participants answered the survey. The age of the participants ranged from 24–63 with the mean age of 46.3 (SD 10.0). The majority, 86% (n=79), of the participants were women and 14% (n=13) were men. The participant length of experience in profession ranged from 0.5-41 years, with the mean of 19.2 (SD 12.0) years. The largest group participating were nurses (n=50), also showing the highest response rate of 54%.

**Table 2:** Participant characteristics and response rate

**Professions**	**Sent out n**	**Answers n (%)**	**% of total answers (n/92)**
Physician	19	9 (47)	10
Nurse (including midwife/other specialities)	103	50 (49)	54
Assistant nurse	43	12 (28)	13
Physiotherapist/Occupational Therapist	18	10 (56)	11
Other *	27	11 (41)	12
Total	210	92 (44)	100

* Others are e.g. dieticians, psychologists, audiologists and administrators.

### Psychometric properties

The Improvement Effectiveness Outcome dimension consists of three items and has a Cronbach’s alpha coefficient of 0.67. The factor loadings were between 0.72-0.86, and the floor/ceiling effect was between 1.1-27.2 percentages. The Internal Improvement Processes dimension consists of eight sub-dimensions with a total of 24 items. Cronbach’s alpha coefficient for the complete dimension was 0.72, and the sub-dimensions had Cronbach’s alphas between 0.23-0.76. For dimensions showing Cronbach’s alpha between 0.60 and 0.70, the number of items needed to reach 0.70 are calculated (Table 3). Factor analysis was conducted at both complete dimension level and at sub-dimension levels. Two sub-dimensions had less than two items and therefore no factor loading or Cronbach’s alpha was calculated. The floor/ceiling effect was between 0–65.2 percentages. The questionnaire factor loadings, Cronbach’s coefficient alpha and % floor/ceiling effect are shown in Table 3.

**Table 3:** Dimensions and items, Cronbach’s coefficient alpha, factor loadings and floor/ceiling effect for the Swedish Improvement Measurement Questionnaire (SIMQ) (n=92 when no other stated)

	**Construct SIMS**	Cronbach’s coefficient alpha * (+/− needed items to reach 0.7 reliability)	Factor loadings calculated at each sub dimension *	**Factor loadings calculated at dimension level**	**Floor/Ceiling effect (%)**
**Item**	**Improvement Effectiveness Outcome (3 items)**	**0.67 (+1)**			
22	Progress satisfaction			0.86	1 / 21
23	Progress meeting expectations			0.77	2 / 4
24	Improvement attains organizational goals			0.72	2 / 27
**Item**	**Internal Improvement processes (24 items)**	**0.72 (−1)**			
	*Improvement Uncertainty (2 items)*	0.28 ^^			
2	Difficulty to know improvement steps		0.77	0.26	0 / 1
7	Frequency difficulty problems arise		0.77	0.39	1 / 46
35	*Resource Scarcity (5 items)*	0.76 (−1)			
a	Competition for finances		0.73	0.20	4 / 46
b	Competition for materials		0.75	0.19	1 / 52
c	Competition for management attention		0.68	0.34	5 / 34
d	Competition for personnel		0.81	0.48	10 / 24
e	Competition for time		0.62	0.19	22 / 4
	*Standardization of Procedures (1 item)*	*			
3	Details of rules and procedures		*	0.49	1 / 3
6	*Decision Influence (4 items )*	0.71 (0)			
a	Deciding on improvement goals (n=91)		0.83	0.65	0 / 38
b	Deciding on work to be done (n=90)		0.88	0.59	0 / 39
c	Deciding on funding (n=64)		0.57	0.11	42 / 4
d	Deciding on personnel recruitment (n=71)		0.73	0.22	23 / 14
	Expectations of Rewards and Sanctions **	0.34 ^^			
	*Individual level (2 items)*	0.60 ( +1)			
15b	Chance of individual reward		0.85	0.41	0 / 46
16b	Chance of individual reprimand		0.85	0.35	0 / 51
	*Group level (2 items)*	0.34 ^^			
15a	Chance of group reward		0.78	0.57	10 / 12
16a	Chance of group reprimand		0.78	0.02	29 / 2
	*Improvement Group Leadership (5 items) ^*	0.66 (+1) (0.76 (−1)) ^			
10	Initiative encouraged		0.80	0.60	1 / 40
11	Members clear about responsibilities		0.52	0.53	0 / 41
12	Emphasis on task		0.88	0.66	1 / 42
13	Leader puts trust in members		0.81	0.61	0 / 55
21	Clear feedback		0.22	0.48	2 / 19
	*Freedom to Express Doubts (1 item)*	*			
14	Freedom to “rock the boat”		*	0.14	2 / 65
	*Learning Encouragement (2 items)*	0.23 ^ ^			
33	Failure not a career blight		0.75	0.22	0 / 34
34	Learning a high organizational priority		0.75	0.37	2 / 5

* In sub-dimension with only one item no factor analyses or Cronbach' s alpha are calculated.

** This subcategory is divided into two because of its divergence.

^ Only four items scale, item 21 deleted.

^^ Sub-dimensions with Cronbach' s alpha below 0.6 no items needed to reach 0.7 reliability are calculated; due to their divergence this is not useful.

### Correlations

The Improvement Effectiveness Outcome item *“Overall, how satisfied are you with the progress that has been made in the work to develop the improvement idea during the past month?”* correlated significantly to three items in the Internal Improvement Processes dimension. Those were: *“How much must your improvement idea compete with other activities within your unit, when it comes to: Time to work with the improvement idea?”* (r=0.34, p=0.029); *“The participants involved in the improvement idea are aware of their individual responsibilities”* (r=0.35, p=0.024); and *“To avoid causing disharmony I often feel I cannot say what I think about the work on the improvement idea”* (r=0.53, p=0.000).

### Background data results

The time spent on working with the improvement idea during the last month ranged from 0 to 80 hours and on average the participants spent 12 (SD 10.6) hours on this work. Most of the time was spent on the participant's own education, mean 3.2 (SD 4.6) hours, followed by time for planning and administration, mean 2.9 (SD 3.3) hours. Least time was spent on acquiring economic funds and resources, mean 0.2 (SD 0.6) hours.

Having prior experience working with improvements was stated by 30% (n=27) “Some”, 27% (n=25) “Quite a bit” and 2% (n=2) “A lot”, while 18% (n=17) answered “None” and 23% (n=21) “A little”. At the same time 50% (n=46) of the respondents stated having “No education” beforehand, 35% (n=32) stated “Participated in courses/training”, 10% (n=9) “University/college-level education” and 5% (n=5) “Other, specify below”. Those answering “Other” had mostly participated in earlier improvement programs.

### Results from the Swedish Improvement Measurement Questionnaire

The dimension “Improvement Effectiveness Outcome” showed that the majority of the respondents were satisfied with their work and what they had accomplished, and they thought that the improvement idea contributed to improve the work at the unit, and that progress was above their expectations. The overall item “How much commitment do you feel toward the improvement idea?” showed a large engagement in the quality improvement initiative: 90% answered “Much/Very much”, 9% “Moderate” and 1% of the participants answered “Very little/Little” (Table 1).

The “Internal Improvement Processes” consist of eight sub-dimensions with a total of 24 items. The five items in “Resource Scarcity” affect the work with the improvement idea differently. A majority of the respondents stated that they had to compete for time to work with the improvement idea while competition for economic resources was mostly stated as “Not at all/A little”. Least competition was about materials, space and equipment. Regarding the item if the methods used supported the work almost half of the respondents answered, “Moderate” (46%). The respondents thought that they could express doubts about the improvement work, and that no one would be punished if they did not achieve the goals. For the dimension “Decision Influence” a high number of respondents stated that they had influence in the work with the improvement idea, such as deciding on measurements and activities, but not regarding resources and colleagues.

### Written free comments

The most common comments were about time; not getting enough time to work with the improvement idea and that it was hard to find time because of regular tasks. There were also comments about manager support, such as wanting help from the manager to plan time for the team to meet. Other comments were about methods of measurements and how to show results. Need for evaluations and assessments to show improvements and distribute achieved results to other colleagues not involved were commented on. Suggestions on improving the initiative were about getting more knowledge and using unit development days to work with improvements.

## Discussion

The aim of this paper was to develop and test an instrument to measure improvement processes and outcomes in Swedish healthcare. A review by Rhydderch et al. [11] shows that there are some instruments tested to validate quality improvements, but none covering all stages required to properly fulfil design and development criteria. We did not find any existing survey that suited our aim, but some of the dimensions in the quite extensive MIS were suitable, and therefore, with permission from its creators, were used as a foundation [8]. The revision was extensive, resulting in a different questionnaire altogether, still consisting of the two dimensions and sub-dimensions from the original survey, although reworded to better fit in the Swedish improvement context measured. The questionnaire was shortened to a large extent. Many of the earlier participants in the focus group and experts consulted agreed that the survey was too comprehensive to start with.

The sub-dimensions in “Internal Improvement Processes” vary greatly, such as items in “Standardization of Procedures”, “Expectations of Rewards and Sanctions” and “Learning Encouragement”. In two sub-dimensions there were only two items, consequently the factor analysis will only measure correlations. The four items in the sub-dimension “Expectations of Rewards and Sanctions” were divided into two parts due to their different focus; two are about rewards and sanctions at an individual level, and the other two at a group level. Doing this division left only two items in each part, making the factor analysis only calculate a correlation. Looking at the two different focuses, individual and group level, this division is arguably necessary. Most of the factor loadings for the items on a sub-dimension level, in dimensions containing of more than two items, were above 0.6, which is considered acceptable [12]. The last item in the sub-dimension “Improvement Group Leadership” loads at 0.22. This item is about feedback from supervisors/improvement support and the other four items are about team leadership and responsibilities. Seeing that feedback is an interesting and important task, the item has not been removed. Although the overall Cronbach’s alpha was 0.72, some of the alpha scores of the sub-dimensions were quite low. Cronbach’s alpha is about internal consistency, and divergence of the items could explain those low values. Another explanation could be the fact that divided into sub-dimensions there are very few items, and calculating Cronbach’s coefficient alpha is problematic with only a few items. Within sub-dimensions there are differences in answer scales, therefore the results in Table 1 are presented in actual frequencies and percentages item by item as well as mean, SD and median [13]. The sub-dimensions were not homogenous within themselves, and therefore the results are shown at item level, and no sum scales on dimension bases can be useful. The items are kept in the questionnaire however because of their nature, telling something important about the improvement processes. Strainer and Norman speak about content validation which is not based on any test or measurements, but on the judgement of experts regarding the substance and content of the items [3]. During the development of the questionnaire, we tried to take this validation into account, first conducting a focus group discussion with former participants and then by consulting an expert in quality management. Between each development step, the authors had recurrent meetings discussing the changes made and the next step to take.

Some items show quite high floor/ceiling effects. Normally this indicates that the scales are too narrow to separate extremes [14]. The possibility to tell something about those extremes doesn’t exist, and the variation of the scale is therefore often found to be too narrow, the variation too limited. In this kind of questionnaire, however, this must not be seen as a problem. If more than 50% of the respondents stated that the leaders trust the team members, this must be considered positive. Probably (and hopefully), a broader scale would not have changed that statement. Another problem is the zeros: if the scale is not fully used, the scale can not be considered a “five-point scale”. Perhaps the variation would not have been greater using a scale with more alternatives. The zeros in the endpoints can not be more then “Absolutely” in the scale ends. Then, one can argue, is there a need to measure if the majority is that positive? This questionnaire is to be used to measure the processes, repeated during the improvement program, and perhaps (and hopefully) some more differentiation will be shown over time. Another possible explanation may be the small sample size. The scales are different, and in some cases the questionnaire probably would have benefited from more consistent scales, although Strainer and Norman argue that it is more important to use words that make sense than to always obtain scale consistency [3].

There were not many significant correlations, but some interesting findings are that the overall satisfaction with the quality improvement initiatives correlates positively to the awareness of individual responsibilities and the feeling of openness, to express what you think about the work. Ekvall [4] describes this in terms of the organisation’s climate, implying a certain degree of openness, commitment, motivation and risk-taking mentality. The more positive the climate is, the more innovative the organisation and the more satisfied the employees will be. He also claims that risk-taking, dynamism and freedom play a role in creating an innovative organisation. In our study the items concerning decision influence did not show this. But the awareness of individual responsibility as well as the item about commitment (even if no correlations were found) can be factors having influence on satisfaction. Kvist et al. [15] also emphasize that people can not be managed to improve; they need to be motivated and encouraged to use their knowledge and abilities in a productive way.

The most common comments in our survey, however, were about lack of time to work with the improvement idea. The respondents thought they needed scheduled time so the whole team could meet and work together. The ordinary daily work always intruded. The time issue is supported by the fact that 52% stated that they had to compete for time to work with the improvement idea. At the same time, most respondents were satisfied with what they had accomplished, and thought their work was useful to the unit and to the healthcare organization. Respondents stated that they had to compete for time, which applies to the comments about lack of time. The least competition was mentioned with respect to economic resources. The fact that 62% stated that they did not have to compete for attention from management (executive level) is interesting. At the same time a recurring comment was about wanting more attention and support from management. Surprisingly there was a positive correlation between the overall satisfaction and the time competition with other activities. Kvist et al. [15] also found that important factors for perceptions of good quality were amount of work, content and influence of development, summarized as possibility to decide about one’s own work. In our study, most participants thought that they could influence the work, which may have contributed to the overall positive experience.

The response rate was 44%. The comparison between the participants showed that nurses were the largest group participating (Table 2). The possible number of participants was 210, but only 92 chose to answer the questionnaire. All we know about those who did not answer are their professions, and comparing to those who did answer, assistant nurses participated the least; only 28% completed the questionnaire. Amongst the other professions about half of the respondents answered, between 41-56% (Table 2). All the professions were represented in this study, to varying degrees. The reason why assistant nurses chose not to participate to such a large extent is not known. Lately, there has been a discussion about decreasing response rates in survey studies in Swedish healthcare [16]. However, the participants in this study were participating in an improvement program, and this was an evaluation of that program, so one might have thought that the motivation to respond should be greater.

### Limitations

The Internal Improvement Processes dimension consists of eight quite divergent sub-dimensions, some of which are only made up of one or two items. Therefore the psychometric properties are below expected values. Four sub-categories, including the divided sanctions and reward dimension, consist of only two items each. Therefore the factor analysis will measure correlations instead. Two other sub-dimensions consist of only one item each, which makes psychometric analyzes impossible. Despite this, it was decided not to remove those items from the questionnaire, because we think they add information about the improvement processes and outcomes we wanted to investigate.

This paper was the first evaluation of the Swedish Improvement Measurement Questionnaire. It is also the first analysis of data following an improvement program using a Collaborative methodology. More data will be collected in this county council quality improvement program, and more analysis of the improvement processes and outcomes in Swedish healthcare settings is forthcoming. Although the questionnaire was developed to fit the Swedish healthcare context, we think that other, similar healthcare systems could make use of the questionnaire, investigating how their improvement efforts are developed. The questionnaire was intended to be quite short, and therefore possible to handle within healthcare. This was an important issue stated by all participants helping us to validate the questionnaire during the different development steps.

## Conclusions

The psychometric property tests, although divergent, suggest initial support for the questionnaire to follow and evaluate quality improvement initiatives in Swedish healthcare settings. The questionnaire would, however, benefit from more development regarding the extent, language and uniform items and standardized measurement scales.

## Competing interests

The authors declare that they have no competing interests. This study is part of postgraduate studies with financial support from Swedish Association of Local Authorities and Regions (SALAR), and the County Council Research and Development Delegation.

## Authors’ contributions

A-CA, ME, K-IP, EI have contributed on the study design and revision of the survey. A-CA conducted the data collection. A-CA and EI have mainly conducted the analysis and drafted the manuscript. All authors have assisted in the interpretations of the results and critically reviewed and approved the final manuscript.

## Pre-publication history

The pre-publication history for this paper can be accessed here:

http://www.biomedcentral.com/1472-6963/13/48/prepub
